# Social networking older adults with mild cognitive impairment: Systematic review protocol on their use of information and communication technology

**DOI:** 10.1371/journal.pone.0302138

**Published:** 2024-05-02

**Authors:** Rongfang Zhan, Elias Mpofu, Gayle Prybutok, Stan Ingman

**Affiliations:** 1 Department of Rehabilitation and Health Services, University of North Texas, Denton, Texas, United States of America; 2 School of Health Sciences, University of Sydney, Camperdown, Australia; 3 Department of Educational Psychology, University of Johannesburg, Johannesburg, South Africa; University of Northumbria at Newcastle: Northumbria University, UNITED KINGDOM

## Abstract

This systematic review will identify and synthesize the emerging evidence on older adults with Mild Cognitive Impairment (MCI) utilizing Information and Communication Technology (ICT) to maintain, restore, or augment social networks. The systematic review will consider the evidence on contextual and personal factors of older adults with MCI and their ICT use for social connectedness. The evidence searches will be implemented in PsycINFO, Academic Search Complete, Medline, PubMed, and manual searches. We shall review articles that were published between January 2010 and October 2023 in English and on Information and Communication Technology utility in social networking among older adults with MCI. The process of article selection will be conducted through title screening, abstract screening; and full article screening, following the Population, Intervention, Control, Outcomes (PICO) criteria. Given that all the studies included in this review are publicly accessible and have already obtained ethical approval from their respective institutions, there is no obligation for us to seek additional ethical clearance for our systematic review. We plan to share the outcomes of the systematic review through online presentations and dissemination within the research community. The findings from this review will identify the extent of empirical evidence on older adults with MCI utilizing ICTs to maintain, restore or augment their social networks. This review will provide evidence for contextual and personal factors in older adults with MCI for the social networks with ICT use. This review will propose practical implications for the effective utilization of ICT by older adults with MCI.

## Introduction

As people age, they tend to lose their social networks, placing them at disproportionate risk for developing cognitive decline and impairment [1,2]. For those aging with or into Mild cognitive impairment (MCI), they are at higher risk for loss of social networks [3,4], perhaps due to lack of social stimulation from interactions with others resulting in social isolation and/or loneliness [5]. Social isolation refers to an objective social reality that lacks connections with social relationships [6,7]. Loneliness refers to the subjective feeling of aloneness or disconnectedness from discrepancy between one’s desire for social connections and their real-life social interactions [8,9].

Mild Cognitive Impairment (MCI) is an intermediate clinical state of cognitive deterioration between normal aging and Alzheimer’s disease [10–12]. Individuals with mild cognitive impairment experience a decline in everyday life cognitive abilities such as memory, thinking, language expression at a higher level than expected for age [13,14], and which may harm their social engagement or social networking. Social networks provide older adults with connections to other people, such as family, friends, acquaintances, and neighbors, through meaningful social interaction [15]. Limited social networks could potentially exacerbate cognitive decline [16–19]. Social networks are beneficial to health functioning in both personal and physical connectivity utilizing information and communication technology tools [20].

Information and Communication Technology (ICT) is fast becoming an everyday tool for usage by older adults, with the historical digital divide diminishing [21,22]. ICT usage encompasses various digital tools and platforms such as computers, smartphones, social media, web-based sites, email, telehealth and so on [23,24]. Use of ICT may assist older adults with MCI to age successfully by maintaining, restoring, and augmenting their social networks. Using information and communication technology, older adults foster positive social relationships, thereby mitigating loneliness and social isolation, and alleviating cognitive decline during the COVID-19 pandemic [25]. Successful aging is being able to maintain physical and mental well-being, social engagement, and overall life satisfaction at older age [26]. For instance, the ownership of smartphones among older adults has experienced a significant surge, increasing from 18% in 2013 to 61% for those aged 65 and above in the year 2021 [27]. Yet, while there exists extensive literature on ICTs usage by older adults, the evidence remains unclear regarding their utilization specifically for social networking purposes, particularly among those with MCI who are at a high-risk population for social isolation and loneliness.

However, within the current digital age, social networks supported by ICT have transcended physical boundaries and seamlessly expanded their influence to encompass the dynamic interactions unfolding on online social sites [28]. But this may vary in unknown ways based on the older adult’s life situations (e.g., living arrangement, geographical area, neighborhood) and personal factors of age, health status, gender et cetera. There is a need to synthesize the evidence of the ICT utility supporting social networking of older adults with MCI for trends that would inform related studies. Our systematic review will synthesize the emerging evidence on Information and Communication Technology utility in social networking among older adults with Mild Cognitive Impairment.

The systematic review we propose will address the following questions:

1. To what extent do older adults with MCI utilize ICTs to maintain, restore or augment their social networks?
2. What is the evidence for contextual and personal factors of older adults with MCI use of ICT for their social networking?

## Methods

### Research design

The systematic review will follow the guidelines of Preferred Reporting Items for Systematic Reviews and Meta-Analysis 2020 [29]. We will use the PRISMA 2020 checklist [29] to ensure a transparent and reliable approach in conducting our systematic review. This protocol is registered on PROSPERO (international prospective register of systematic reviews) (registration number CRD42023469950).

### Eligibility criteria

We will specify the inclusion and exclusion criteria for the review, with reference to the study characteristics including Population, Intervention, Control, Outcomes (PICO), study design, setting, time frame, and the report characteristics such as the years considered, publication status, and language (Table 1).

**Table 1:** Criteria for inclusion and exclusion of studies in the review.

Category	Inclusion	Exclusion
Population	Outlined the perspectives of older adults (65 years or older) with mild cognitive impairment. Defined MCI as ‘a deficit in memory that does not significantly impact daily functioning’.	Other age groups such as younger adults or individuals without MCI. Without clear diagnostic criteria for MCI and Severe cognitive impairment that significantly impacts daily functioning.
Interventions	Discuss the use of Information and Communication Technology such as smartphone, computer, social media platforms such as Facebook and twitter, and internet-based tools to support social activities among older adults with MCI in social participation.	Daily assistive technology
Control	ICT usage and. Different types of systems.	Traditional in-person social networking
Outcomes	ICT supported social networking, including measures of any changes to the level of social networks. Self-reported assessments of social life using ICT.	
Study design	Peer reviewed papers, Conference papers	book chapters or review articles.
Settings	No limit such as community setting, nursing home, assisted living	
Timing	Minimum duration of the ICT usage: 2 months	
Date of search	Searches will start from January 2010 to October 2023	
Publication language	English	Other languages

Abbreviations: MCI mild cognitive impairment, ICT information and communication technology.

#### Population

We will include for the systematic review studies on older adults who are 65 years or older with MCI and exclude populations with severe memory functions such as dementia and Alzheimer’s disease. Rubinow [30] defined that “age 65 is generally set at the threshold of old age since it is at the period of life that the rates for sickness and death begin to show a marked increase over those of the earlier years.”

#### Types of ICT usage

The systemic review will examine the evidence from studies that specify the use of ICT in social networks. ICT usage includes any form of communication with at least one other person. Due to the concentration of the review on the implementations of ICT, other types of technologies such as daily assistive technology will be excluded.

#### Types of control and comparison

Control and comparison refer to the study elements on ICT usage for social lives by older adults. The comparator will include three scopes based on the feature of each included study: 1) ICT usage and no ICT usage. We will compare the outcomes of social networks in older adults with MCI between how they use ICT and don’t use ICT; 2) ICT usage and traditional in-person social intervention. We will compare the evidence from studies using ICT and traditional methods such as only in-person social support groups in older adults with MCI; 3) different types of ICT usage. The review will compare the effectiveness of different types of ICT in included studies such as smartphone, tablets, and social media or technologically based cognitive training programs on social networks. Additionally, we also explore the effectiveness of various online platforms, such as social media platforms and other web-based applications, in facilitating social networks among older adults.

#### Types of outcome measures

The outcome measures will assess the evidence on the level of social networks among older adults with MCI by their ICT usage. We will assess the evidence on usage to maintain existing social networks, to restore previous social networks, and/or to form new social networks. We also will examine the evidence from studies that utilized standardized cognitive assessment tools and self-reported assessments of cognitive functioning and social networks using ICT.

#### Setting

The systematic review will include studies on older adults in community living as well as those in assisted living and able to use Information and Communication Technology. Institutions such as assisted living, retirement homes, hospitals or nursing homes are included. We will exclude studies that are implemented in memory care facilities taking care of older adults with dementia and Alzheimer’s disease.

#### Types of study designs

Eligible for the systematic review inclusion are peer reviewed studies, nonrandomized controlled trials, randomized controlled trials, case-control studies, and prospective controlled cohort studies.

#### Timing

For ICT intervention studies, we will include those that implemented a minimum duration of at least two months.

#### Date of search

The inclusion of the date of the literature will start from January 2010 to October 2023.

#### Publication language

The proposed systematic review will focus on English published studies. We will exclude the studies that are published in other languages.

### Information sources

Studies will be systematically identified through structured searches from key databases: PsycINFO, Academic Search Complete, Medline, and PubMed. In addition, we will conduct manual searches by searching relevant studies in Google Scholar.

### Search strategy

Included studies are those published between January 2010 and October 2023. Key concepts of the proposed systematic review will be ‘older adults’, ‘mild cognitive impairment’, and ‘information communication technology’, ‘social networks’, ‘usage status’ and we extend our search terms listed below (Table 2).

**Table 2:** Final search strategy on key concepts and search terms.

	Key Concepts		Search Terms
	Mild Cognitive Impairment	OR	“Cognitive decline” OR “cognitive impairment, mild “OR “MCI” OR “Preclinical Alzheimer’s Disease “OR “cognitive dysfunction, mild” OR “Memory Impairment” OR “cognitive deficit” OR “cognitive deterioration” OR “cognitive aging”
AND	Older adults	OR	“Older adult” OR elderly OR aging OR geriatric * OR “older people” OR “Aged 65” OR over 65” OR “65 years” OR “65<” OR “65yr OR “65” OR “elderly.” OR senior * OR aged or older elder or geriatric* or “elderly people” OR “older people”
AND	Information and Communication Technology	OR	“Information and Communication Technology” OR “ICT*” OR “Digital technology” OR “Digital communication” OR “Technological communication” OR “Digital media” OR “Online communication” OR “Internet technology” OR “Virtual communication” OR “Web-based communication” OR “Telecommunication”
AND	Social networks	OR	“Social networking” OR “social interaction”, OR “social ties” OR “social contact” OR “social connection”
AND	Usage status	OR	“Maintain” OR “Restore” OR “Augment”

* This symbol signifies unlimited searches of diverse forms of a word, created by attaching various suffixes.

### Selection process and data collection process

The process of article selection will be conducted through three consecutive steps: 1) title screening; 2) abstract screening; and 3) full article screening. All extracted articles will be stored in a RefWorks database. The duplication elimination procedure will be handled in RefWorks as well. The first author reviewer will employ text extraction techniques by using Python to retrieve all titles and abstracts saved in a PDF, and then two reviewers will independently review all titles and abstracts in the PDF to further identify each study for eligibility against our inclusion and exclusion criteria. After identifying the included titles and abstracts, we will retrieve and review the full text of selected titles. Two reviewers will independently review the full-text articles, with any discrepancies addressed until a unanimous agreement is reached.

The systematic review process will be illustrated visually in the PRISMA 2020 flow diagram that is a version for new systematic reviews which included searches of databases, registers, and other sources [29].

For ease of interpreting included studies, we will create data abstraction tables to classify extracted study information from each study. The tables will include the study characteristics (e.g., author, publication year), participant characteristics (e.g., age, gender, race, sample size), types of ICT usage, study conducted in the USA, methods, and findings. The first author and second author reviewer will independently review and verify the information included in the evidence tables for accuracy. Any discrepancies in the results will be resolved through discussion with a third reviewer, as necessary.

### Data items

We will identify the types of ICT usage and present the setting, method, participant characteristics and findings of each included study.

### Risk of bias assessment

Two independent reviewers will independently access the risk bias for data information extracted from the studies. Any discrepancy of information between two reviewers will be solved by discussing with third and fourth team members. To ensure the methodological quality of each included study, we will appraise the studies based on the Assessing the Methodological Quality of Systematic Reviews (AMSTAR) tool. We will use the Cochrane Risk of Bias Tool (CRB) for appraisal of the randomized controlled trials and the Risk of Bias in Non-randomized Studies of Interventions (ROBINS-I) tool for verifying nonrandomized studies.

### Data synthesis

The proposed systematic review will incorporate a descriptive numerical summary for quantitative studies and a qualitative thematic synthesis for qualitative studies [31]. If we find comparisons of interest on included studies, we will conduct a descriptive numerical summary that will structure and present main characterizations of included studies including the types of ICT intervention, types of study design, year of publication, characteristics of the participants, settings where studies were conducted, and other features. For in-depth understanding, the topic of the proposed systematic review implements a qualitative thematic synthesis. The qualitative thematic synthesis will be conducted to identify the common themes or patterns of meaning across the included studies [31]. Further, we focus on social networking activity and participation for those suffering with MCI supported by ICT.

### Ethics and dissemination

The ethical approval status of each included study will be appraised by two independent reviewers. Since all included studies are publicly available and approved by their initial studies, we are not required to provide ethical approval for our systematic review. The findings of the systematic review will be presented online and disseminated to the research community.

## Results

Synthesizing the evidence on older adults with MCI of ICT usage for social networking is critical for researchers, stakeholders, practitioners, and policymakers to improve ICT services for the social well-being of older adults with MCI. Finding may contribute to the further ICT innovations in older adults with MCI. We shall present the results in three tables: evidence trends across the total sample of all studies, contextual factors, and personal factors.

## Discussion

This systematic review protocol provides an outline and framework for further systematic review that fills the research gap in ICT utilization in social networks by older adults with MCI. To our knowledge, there is no previous review trending evidence on ICT utility for social networking by older adults with MCI. Particularly, we will provide a holistic framework for older adults with MCI’s use of ICT to maintain, restore and/or augment their social networks. We shall discuss the findings for trends in the evidence on ICT-supported social connectedness rapidly becoming the new normal for social interactions with MCI, important to older adults maintaining, restoring, and augmenting their social networking.

## Supporting information

S1 Checklist PRISMA-P (Preferred Reporting Items for Systematic review and Meta-Analysis Protocols) 2015 checklist: Recommended items to address in a systematic review protocol*. (DOCX)
