# Supplementary material for: Social networking older adults with mild cognitive impairment: Systematic review protocol on their use of information and communication technology
Source: PLoS One. 2024 May 2;19(5):e0302138. doi: 10.1371/journal.pone.0302138 (PMC11065287; doi:10.1371/journal.pone.0302138)
Supplement: S1 Checklist — (DOCX) [file pone.0302138.s001.docx]

**PRISMA-P (Preferred Reporting Items for Systematic review and Meta-Analysis Protocols) 2015 checklist: recommended items to address in a systematic review protocol***

| **Section and topic** | **Item No** | **Checklist item** |
| --- | --- | --- |
| **ADMINISTRATIVE INFORMATION** | | |
| Title: |  |  |
| Identification | 1a | This report is a protocol of a systematic review |
| Update | 1b | It is a new protocol and not an update of a previous systematic review **Yes** |
| Registration | 2 | PROSPERO registration number CRD42023469950 |
| Authors: |  |  |
| Contact | 3a | Provide name e-mail address of all protocol authors.  Rongfang Zhan^1^ [Rongfang.Zhan@unt.edu](mailto:Rongfang.Zhan@unt.edu)  Elias Mpofu^1,2,3^ [elias.mpofu@unt.edu](mailto:elias.mpofu@unt.edu)  Gayle Prybutok^1^ [gayle.prybutok@unt.edu](mailto:gayle.prybutok@unt.edu)  Stan Ingman^1^ [stan.ingman@unt.edu](mailto:stan.ingman@unt.edu)  , institutional affiliation,  ^1^Department of Rehabilitation and Health Services, University of North Texas,  Denton, TX, United States  ^2^School of Health Sciences, University of Sydney, Australia  ^3^Department of Educational Psychology, University of Johannesburg, South Africa  provide physical mailing address of corresponding author  * Corresponding author  E-mail: Rongfang.Zhan@unt.edu (RZ)  Chilton Hall 410 Avenue C, Suite 116 Denton, Texas, 76201 |
| Contributions | 3b | Describe contributions of protocol authors and identify the guarantor of the review Author Rongfang Zhan work on study conception and design, data extraction and analysis, and manuscript preparation. Author Elias Mpofu, conceptualization, verification of the data extraction and analysis, and supervises the study. Author Gayle Prybutok and Stan Ingman co-supervise and overview the study. Author Elias Mpofu is a guarantor of the protocol. |
| Amendments | 4 | If the protocol represents an amendment of a previously completed or published protocol, identify as such and list changes; otherwise, state plan for documenting important protocol amendments Not Applicable |
| Support: |  |  |
| Sources | 5a | Indicate sources of financial or other support for the review Graduate Student Research Assistantship |
| Sponsor | 5b | Provide name for the review funder and/or sponsor Rehabilitation and Health Services |
| Role of sponsor or funder | 5c | Describe roles of funder(s), sponsor(s), and/or institution(s), if any, in developing the protocol Provided computer equipment for searches and subject librarian support |
| **INTRODUCTION** | | |
| Rationale | 6 | Describe the rationale for the review in the context of what is already known: Current digital age, social networks supported by ICT have transcended physical boundaries and seamlessly expanded their influence to encompass the dynamic interactions unfolding on online social sites. Yet, we do not know the trending evidence on how older adults with MCI utilize ICTs to maintain, restore or augment their social networks |
| Objectives | 7 | Provide an explicit statement of the question(s) the review will address with reference to participants, interventions, comparators, and outcomes (PICO) 1) To what extent do older adults with MCI utilize ICTs to maintain, restore or augment their social networks? 2) What is the evidence for contextual and personal factors of older adults with MCI use of ICT for their social networking? |
| **METHODS** | | |
| Eligibility criteria | 8 | Specify the study characteristics (such as PICO, study design, setting, time frame) and report characteristics (such as years considered, language, publication status) to be used as criteria for eligibility for the review   \| **Category** \| **Inclusion** \| **Exclusion** \| \| --- \| --- \| --- \| \| Population \| Outlined the perspectives of older adults (65 years or older) with mild cognitive impairment.  Defined MCI as ‘a deficit in memory that does not significantly impact daily functioning’. \| Other age groups such as younger adults or individuals without MCI.         Without clear diagnostic criteria for MCI and Severe cognitive impairment that significantly impacts daily functioning. \| \| Interventions \| Discuss the use of Information and Communication Technology such as smartphone, computer, and internet-based tools to support social activities among older adults with MCI in social participation. \| Daily assistive technology \| \| Control \| ICT usage and.  Different types of systems. \| No ICT usage for social networking  Traditional in-person social networking \| \| Outcomes \| ICT supported social networking, restoration and increased the level of social networks.  Self-reported assessments of social life using ICT \|  \| \| Study design \| Peer reviewed papers \| Conference paper, book chapters or review articles. \| \| Settings \| No limit such as community setting, nursing home, assisted living \|  \| \| Timing \| Minimum duration of the ICT usage: 2 months \|  \| \| Date of search \| Searches will start from January 2010 to October 2023 \|  \| \| Publication language \| English \| Other languages \| |
| Information sources | 9 | Describe all intended information sources (such as electronic databases, contact with study authors, trial registers or other grey literature sources) with planned dates of coverage.  Studies will be systematically identified through structured searches from key databases: PsycINFO, Academic Search Complete, Medline, Web of Science, and PubMed. In addition, we will conduct manual searches by searching relevant studies in Google Scholar. Searches will start from January 2010 to October 2023 |
| Search strategy | 10 | Present draft of search strategy to be used for at least one electronic database, including planned limits, such that it could be repeated.  Included studies are those published between January 2010 and October 2023. Key concepts of the proposed systematic review will be ‘older adults’, ‘mild cognitive impairment’, and ‘information communication technology’, ‘social networks’, ‘usage status’ and we extend our search terms listed IN Table below (Table 2).   \|  \| **Key Concepts** \|  \| **Search Terms** \| \| --- \| --- \| --- \| --- \| \|  \| Mild Cognitive Impairment \| OR \| “Cognitive decline” OR “cognitive impairment, mild “OR “MCI” OR “Preclinical Alzheimer's Disease “OR “cognitive dysfunction, mild” OR “Memory Impairment” OR “cognitive deficit” OR “cognitive deterioration” OR “cognitive aging” \| \| AND \| Older adults \| OR \| “Older adult” OR elderly OR aging OR geriatric * OR “older people” OR  “Aged 65” OR 65+ OR “elderly”.  OR senior * OR aged or older  elder or geriatric* or “elderly people”  OR “older people” \| \| AND \| Information and Communication Technology \| OR \| “Information and Communication Technology” OR “ICT*” OR “Digital technology” OR “Digital communication” OR “Technological communication” OR “Digital media” OR “mild cognitive impairment” OR “Online communication”  OR “Internet technology” OR “Virtual communication” OR “Web-based communication” OR “Telecommunication” \| \| AND \| Social networks \| OR \| “Social networking” OR “social interaction”, OR “social ties” OR  “social contact” \| \| AND \| Usage status \| OR \| “Maintain” OR “Restore” OR “Augment” \|   * *This symbol signifies unlimited searches of diverse forms of a word, created by attaching various suffixes.* |
| Study records: |  |  |
| Data management | 11a | Describe the mechanism(s) that will be used to manage records and data throughout the review.  All extracted articles will be stored in a RefWorks database. The duplication elimination procedure will be handled in RefWorks as well. The first author reviewer will employ text extraction techniques by using Python to retrieve all titles and abstracts saved in a PDF, and then two reviewers will independently review all titles and abstracts in the PDF to further identify each study for eligibility against our inclusion and exclusion criteria. After identifying the included titles and abstracts, we will retrieve and review the full text of selected titles. |
| Selection process | 11b | State the process that will be used for selecting studies (such as two independent reviewers) through each phase of the review (that is, screening, eligibility and inclusion in meta-analysis)  The process of article selection will be conducted through three consecutive steps: 1) title screening; 2) abstract screening; and 3) full article screening. Two reviewers (Rongfang Zhan and Elias Mpofu) will independently review the full-text articles, with any discrepancies addressed until consensus is reached. |
| Data collection process | 11c | Describe planned method of extracting data from reports (such as piloting forms, done independently, in duplicate), any processes for obtaining and confirming data from investigators The systematic review process will be illustrated visually in the PRISMA 2020 flow diagram that is a version for new systematic reviews which included searches of databases, registers, and other sources (Page et al., 2021).  For ease of interpreting included studies, we will create data abstraction tables to classify extracted study information from each study. The tables will include the study characteristics (e.g., author, publication year), participant characteristics (e.g., age, gender, race, sample size), types of ICT usage, study conducted in the USA, methods, and findings. The first author and second author reviewer will independently review and verify the information included in the evidence tables for accuracy. Any discrepancies in the results will be resolved through discussion with a third reviewer, as necessary. |
| Data items | 12 | List and define all variables for which data will be sought (such as PICO items, funding sources), any pre-planned data assumptions and simplifications We will identify the types of ICT usage and present the setting, method, participant characteristics and findings of each included study. |
| Outcomes and prioritization | 13 | List and define all outcomes for which data will be sought, including prioritisation of main and additional outcomes, with rationale. The outcome measures will assess the evidence on the level of social networks among older adults with MCI by their ICT usage. We will assess the evidence on usage to maintain existing social networks, to restore previous social networks, and/or to form new social networks. We also will examine the evidence from studies that utilised standardised cognitive assessment tools and self-reported assessments of cognitive functioning and social networks using ICT. |
| Risk of bias in individual studies | 14 | Describe anticipated methods for assessing risk of bias of individual studies, including whether this will be done at the outcome or study level, or both; state how this information will be used in data synthesis Two independent reviewers will independently assess the risk bias for data information extracted from the studies. Any discrepancy of information between two reviewers will be solved by discussing with third and fourth team members. To ensure the methodological quality of each included study, we will appraise the studies based on the Assessing the Methodological Quality of Systematic Reviews (AMSTAR) tool. We will use the Cochrane Risk of Bias Tool (CRB) for appraisal of the randomised controlled trials and the Risk of Bias in Non-randomized Studies of Interventions (ROBINS-I) tool for verifying nonrandomized studies. |
| Data synthesis | 15a | Describe criteria under which study data will be quantitatively synthesised. The proposed systematic review will incorporate a descriptive numerical summary for quantitative studies and a qualitative thematic synthesis for qualitative studies (Levac et al., 2010). |
|  | 15b | If data are appropriate for quantitative synthesis, describe planned summary measures, methods of handling data and methods of combining data from studies, including any planned exploration of consistency (such as I^2^, Kendall’s τ) If we find comparisons of interest on included studies, we will conduct a descriptive numerical summary that will structure and present main characterizations of included studies including the types of ICT intervention, types of study design, year of publication, characteristics of the participants, settings where studies were conducted, and other features. |
|  | 15c | Describe any proposed additional analyses (such as sensitivity or subgroup analyses, meta-regression)  No additional analyses |
|  | 15d | If quantitative synthesis is not appropriate, describe the type of summary planned for in-depth understanding, the topic of the proposed systematic review implements a qualitative thematic synthesis. The qualitative thematic synthesis will be conducted to identify the common themes or patterns of meaning across the included studies (Levac et al., 2010). Further, we focus on social networking activity and participation for those suffering with MCI supported by ICT. |
| Meta-bias(es) | 16 | Specify any planned assessment of meta-bias(es) (such as publication bias across studies, selective reporting within studies) This is not a Meta analysis |
| Confidence in cumulative evidence | 17 | Describe how the strength of the body of evidence will be assessed (such as GRADE) . We will apply the GRADE levels of high, moderate, low, or insufficient to qualify the strength of the evidence. |

*** It is strongly recommended that this checklist be read in conjunction with the PRISMA-P Explanation and Elaboration (cite when available) for important clarification on the items. Amendments to a review protocol should be tracked and dated. The copyright for PRISMA-P (including checklist) is held by the PRISMA-P Group and is distributed under a Creative Commons Attribution Licence 4.0.**

*From: Shamseer L, Moher D, Clarke M, Ghersi D, Liberati A, Petticrew M, Shekelle P, Stewart L, PRISMA-P Group. Preferred reporting items for systematic review and meta-analysis protocols (PRISMA-P) 2015: elaboration and explanation. BMJ. 2015 Jan 2;349(jan02 1):g7647.*
